# Sex difference in the association between pathological albuminuria and subclinical atherosclerosis: insights from the I-Lan longitudinal aging study

**DOI:** 10.18632/aging.204331

**Published:** 2022-10-11

**Authors:** Ya-Wen Lu, Chun-Chin Chang, Ruey-Hsing Chou, Yi-Lin Tsai, Li-Kuo Liu, Liang-Kung Chen, Po-Hsun Huang, Shing-Jong Lin

**Affiliations:** 1 Division of Cardiology, Department of Medicine, Taichung Veterans General Hospital, Taichung, Taiwan; 2 Cardiovascular Research Center, National Yang Ming Chiao Tung University, Taipei, Taiwan; 3 Institute of Clinical Medicine, National Yang Ming Chiao Tung University, Taipei, Taiwan; 4 Division of Cardiology, Department of Medicine, Taipei Veterans General Hospital, Taipei, Taiwan; 5 Department of Critical Care Medicine, Taipei Veterans General Hospital, Taipei, Taiwan; 6 Center for Geriatrics and Gerontology, Taipei Veterans General Hospital, Taipei, Taiwan; 7 Aging and Health Research Center, National Yang Ming Chiao Tung University, Taipei, Taiwan; 8 Institute of Public Health, National Yang Ming Chiao Tung University, Taipei, Taiwan; 9 Taipei Municipal Gan-Dau Hospital, Taipei, Taiwan; 10 Taipei Heart Institute, Taipei Medical University, Taipei, Taiwan; 11 Division of Cardiology, Heart Center, Cheng-Hsin General Hospital, Taipei, Taiwan

**Keywords:** albuminuria, microalbuminuria, carotid intima-media thickness, subclinical atherosclerosis, sex disparity

## Abstract

Background: Pathological albuminuria (PAU) (urinary albumin creatinine ratio [UACR] ≥30 mg/g) is an independent risk factor of cardiovascular disease. PAU is more prevalent in men than women. We aimed to compare the association of PAU and the early phase of subclinical atherosclerosis (SA) between sexes.

Methods: 1228 subjects aged 50–90 years were stratified by sex and UACR (normal or PAU). SA was defined as mean carotid intima-media thickness ≥75th percentile of the cohort. Demographics and SA prevalence were compared between groups. Multivariate logistic regression was performed to assess the relationship between PAU and SA.

Results: Both men and women with PAU had increased prevalence of hypertension, anti-hypertensive therapy, and metabolic syndrome than controls. Men with PAU were older and had greater waist circumference and total body fat percentage. Sex disparity was observed in associations between waist-to-height ratio, total body fat, and UACR. After adjusting for traditional risk factors, multivariate logistic regression disclosed that PAU was independently associated with SA in men (adjusted odds ratio 1.867, 95% CI 1.066–3.210) but not in women.

Conclusion: The relationship of PAU and SA differed between sexes. This result may highlight the need for sex-specific risk management strategies to prevent atherosclerosis.

## INTRODUCTION

Cardiovascular disease (CVD) is the leading global cause of death [1]. Age, sex, hypertension (HTN), dyslipidemia, hyperglycemia, obesity, and smoking are significant determinants of incident CVD in the general population [2].

Albuminuria (AU), defined as urine albumin-to-creatinine ratio (UACR) more than 30 mg/g, is recognized as the earliest sign of not only vascular disease but also of renal dysfunction [3, 4]. Although 24-hour urine protein collection is the gold-standard urinary albumin excretion assay, it is cumbersome for patients and is unreliable due to under or over collection and laboratory processing methods [5]. MA can be diagnosed more easily by UACR determinations [6, 7]. Increased UACR has been associated with cardiovascular morbidity and mortality and incident strokes in diabetics [8]. Furthermore, MA is an independent risk factor of coronary heart disease, peripheral vascular disease, and stroke in non-diabetics [9, 10]. Although increased UACR has been associated with maladaptive carotid remodeling that may predispose to stroke [11]; and may result from multiple cardiovascular risk factors such as HTN, age, obesity, and dyslipidemia; the pathophysiologic links of MA to the above diseases are still unknown [12].

Carotid intima-media thickening (CIMT) is an accepted ultrasonographic marker of early subclinical atherosclerosis (SA) and may be a predictive factor of incident CVD [13, 14]. Well-known determinants of CIMT are age, HTN, and sex. Females have exhibited lower CIMT than males in numerous studies, including the Gutenberg-Heart Study in Germany [15], the Atherosclerosis Risk in Communities study [16], and the Suita study of Japanese subjects [17].

Albuminuria has been associated with CIMT. A significant association of urinary albumin excretion and CIMT was observed in hypertensive men with diabetes mellitus (DM) [18]. However, MA was unrelated to CIMT in hypertensive men without DM [19]. Clinical investigations regarding a differential sex-based association of pathological albuminuria (PAU) (UACR ≥30 mg/g) on SA have been limited. This study aimed to determine whether PAU is associated with the early phase of SA, and whether such an association may be sex-determined, especially in relatively healthy populations.

## RESULTS

A total of 1228 participants (46.0% women; mean age 62.56 ± 8.77 years) without coronary artery disease (CAD), DM, or chronic kidney disease (CKD) were investigated. In women, there were no differences of age, body mass index (BMI), waist circumference, waist-to-height ratio, or prevalence of smoking between subjects with or without PAU. In contrast, men with PAU were older and exhibited elevated waist circumference and an increased prevalence of waist circumference >90 cm (a metabolic syndrome criterion) compared to those with normal AU. Significantly higher prevalence rates of HTN and anti-HTN therapy were observed in both women and men with PAU. However, women had more elevated total body fat than men (37.1% vs. 24.8% respectively; *p* < 0.001). Men with PAU had significantly higher total body fat than those with normal AU (26.48 ± 7.42 vs. 24.52 ± 6.46; *p* = 0.027).

Total serum cholesterol, low-density lipoprotein cholesterol (LDL), high-density lipoprotein cholesterol (HDL), hemoglobin A1c, uric acid, and creatinine values were similar between men and women. PAU was associated with higher fasting glucose in women but not in men. Men with PAU exhibited higher triglyceride levels and lower estimated glomerular filtration rate (eGFR). PAU was associated with substantially increased mean CIMT and higher prevalence of SA (CIMT ≥0.75 mm) in men, but not in women (Table 1).

**Table 1:** Baseline characteristics according to UACR ≥ 30 mg/g or not stratified by gender.

	**Female (*n* = 655)**			**Male (573)**		
	**UACR <30 mg/g *n* = 565**	**UACR ≥30 mg/g *n* = 90**	***p* value**	**UACR <30 mg/g *n* = 485**	**UACR ≥30 mg/g *n* = 88**	***p* value**
Age (years)	59.9 (54.95–67.75)	60.7 (55.30–69.45)	0.501	61.1 (55.3–70.0)	65.1 (58.28–74.78)	0.001
BMI	23.92 (21.90–26.3)	24.25 (22.18–27.24)	0.303	24.42 (22.34–26.48)	25.33 (22.47–27.34)	0.087
Waist circumference	80.0 (74.0–86.5)	81.5 (75.0–87.13)	0.496	86 (80.5–91.75)	88.75 (82.88–94.0)	0.007
Waist-to-height ratio	0.53 ± 0.067	0.53 ± 0.063	0.351	0.52 ± 0.050	0.54 ± 0.054	<0.001
Smoking (%)	17 (3.0)	3 (3.3)	0.747	160 (33.0)	34 (38.6)	0.328
**Underlying disease**						
Hypertension (%)	161 (28.5)	46 (51.1)	<0.001	147 (30.3)	42 (47.7)	0.002
Anti-Hypertensive agents (%)	73 (12.9)	25 (27.8)	0.001	77 (15.9)	23 (26.1)	0.031
Dyslipidemia	29 (5.1)	9 (10.0)	0.085	16 (3.3)	4 (4.5)	0.529
Statin	26 (4.6)	9 (10.0)	0.043	14 (2.9)	3 (3.4)	0.734
Metabolic syndrome (%)	136 (24.1)	32 (35.6)	0.027	101 (20.8)	30 (34.1)	0.009
Metabolic syndrome_BS	131 (23.2)	27 (30.0)	0.184	133 (27.4)	33 (37.5)	0.073
Metabolic syndrome_BP	301 (53.3)	66 (73.3)	<0.001	281 (57.9)	68 (77.3)	0.001
Metabolic syndrome_HDL	117 (20.7)	26 (28.9)	0.098	67 (13.8)	15 (17.0)	0.411
Metabolic syndrome_TG	86 (15.2)	17 (18.9)	0.354	118 (24.3)	23 (26.1)	0.689
Metabolic syndrome_WC	297 (52.6)	52 (57.8)	0.366	164 (33.8)	41 (46.6)	0.029
Obesity (%)	116 (20.5)	23 (25.6)	0.270	94 (19.4)	24 (27.3)	0.114
Body total fat % (DXA)	37.17 ± 6.01	36.61 ± 7.58	0.505	24.52 ± 6.46	26.48 ± 7.42	0.027
**Laboratory data**						
Total Cholesterol (mg/dl)	205 (182–225.5)	199 (170.75–227.25)	0.181	192 (170.5–215.0)	191.5 (168.8–212.3)	0.979
HDL (mg/dl)	58 (51–69)	56 (47–67.25)	0.141	50 (43–57)	49.5 (42–57)	0.873
LDL (mg/dl)	122 (101–142.5)	118.5 (98–145.75)	0.484	119 (99–142)	122 (105.3–134)	0.550
Fasting glucose (mg/dl)	93 (87–99)	94.5 (89–101.25)	0.028	94 (89–100)	96 (89–104)	0.136
HbA1c (%)	5.8 (5.6–6.0)	5.8 (5.58–6.10)	0.197	5.7 (5.4–5.9)	5.8 (5.5–6.0)	0.068
Uric acid	5.0 (4.4–5.8)	5.1 (4.3–5.9)	0.836	6.4 (5.5–7.2)	6.4 (5.4–7.4)	0.809
Triglyceride (mg/dl)	94 (70–131)	108.5 (79–137.75)	0.056	102 (74–148)	118 (90–151.5)	0.020
Creatinine (mg/dl)	0.67 (0.60–0.75)	0.65 (0.58–0.74)	0.223	0.89 (0.80–1.00)	0.90 (0.77–1.01)	0.677
eGFR (ml/min/1.73 m^2^)	94.34 (85.81–100.33)	94.98 (87.13–101.23)	0.525	89.49 (79.97–95.98)	86.66 (75.10–96.19)	0.030
UACR	8.09 (5.54–12.97)	55.01 (36.78–97.44)	<0.001	6.06 (3.76–10.40)	47.89 (36.15–72.67)	<0.001
Mean cIMT (mm)	0.6 (0.6–0.7)	0.65 (0.6–0.7)	0.445	0.7 (0.6–0.75)	0.75 (0.66–0.90)	<0.001
cIMT ≥ 0.75 mm	129 (22.8)	16 (17.8)	0.339	155 (32.0)	45 (51.1)	0.001

Values are median (25th–75th percentile) or *n* (%). Abbreviations: BMI: body mass index; DM: diabetes mellitus; HDL: high density lipoprotein; LDL: low density lipoprotein; HbA1c: hemoglobin A1c; eGFR: estimated glomerular filtration rate; UACR: urine albumin creatinine ratio; cIMT: carotid intima-media thickness.

**Figure 1:**
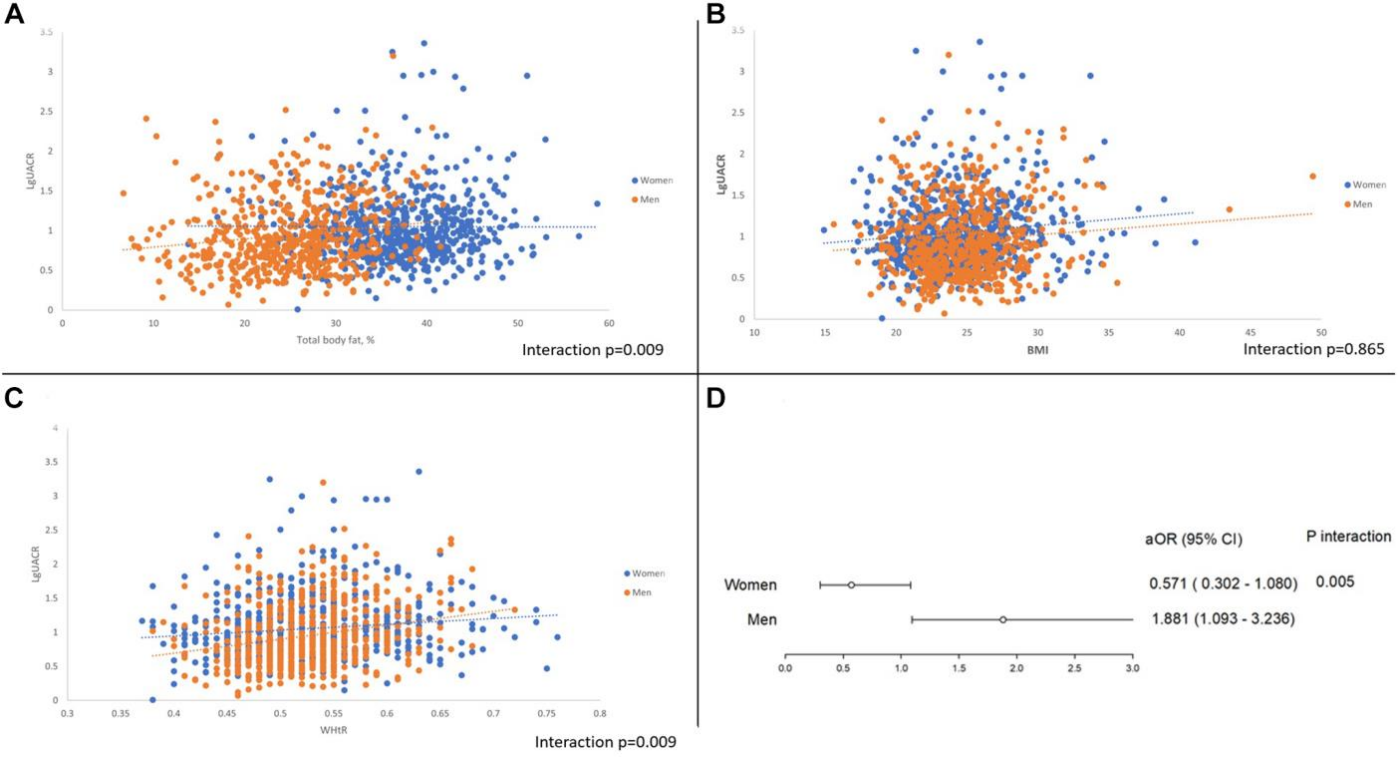
(**A**) Gender disparity between UACR and total body fat percentage. (**B**) Gender disparity between UACR and BMI. (**C**) Gender disparity between UACR and Waist to height ratio. (**D**) Pathological albuminuria and the adjusted odds ratio of subclinical atherosclerosis between sex.

Table 2 shows the correlation coefficients of CIMT and UACR with other variables in women and men. Age, HTN, waist circumference, and waist-to-height ratio were positively correlated with CIMT and UACR in both groups. Obesity and higher BMI were associated with CIMT and UACR only in women, while higher total body fat percentage was positively related to CIMT and UACR in men. Smoking, total serum cholesterol, and low-density cholesterol were unassociated with CIMT or UACR in men and women. Simple linear correlations between UACR and other CV risk factors, including total body fat percentage, BMI, and waist to height ratio by sex are displayed by scatter plots in Figure 1A–1C.

**Table 2:** Correlation coefficients of UACR and the carotid intima-media thickness with other cardiovascular risk factors in the whole population.

**Women**	**cIMT**	**UACR**
Age	0.348^**^	0.151^**^
Smoking	0.044	0.015
Hypertension	0.193^**^	0.181^**^
Dyslipidemia	0.033	0.098^*^
Metabolic syndrome	0.147^**^	0.157^**^
Waist	0.164^**^	0.104^**^
BMI	0.120^**^	0.112^**^
Obesity	0.109^**^	0.135^**^
Total body fat percentage	0.046	−0.017
Waist to height ratio	0.188^**^	0.146^**^
eGFR	−0.260^**^	−0.017
Total Cholesterol	−0.029	−0.045
TG	0.091^*^	0.092^*^
LDL	−0.002	−0.014
HDL	−0.090^*^	−0.060
HbA1c	0.081^*^	0.080^*^
Uric acid	0.134^**^	0.033
UACR	0.093^*^	–
**Men**	**cIMT**	**UACR**
Age	0.390^**^	0.259^**^
Smoking	0.025	0.061
Hypertension	0.160^**^	0.177^**^
Dyslipidemia	0.001	0.083^*^
Metabolic syndrome	0.066	0.114^**^
Waist	0.149^**^	0.164^**^
BMI	0.078	0.065
Obesity	0.004	0.045
Total body fat percentage	0.118^**^	0.154^**^
Waist to height ratio	0.212^**^	0.225^**^
eGFR	−0.220^**^	−0.124^**^
Total Cholesterol	−0.024	0.044
TG	0.015	0.111^**^
LDL	0.041	0.052
HDL	−0.092^*^	−0.015
HbA1c	0.070	0.128^**^
Uric acid	0.067	0.030
UACR	0.187^**^	–

^*^Correlation is significant at the 0.05 level (2-tailed). ^**^Correlation is significant at the 0.01 level (2 tailed). Abbreviations: BMI: body mass index; HDL: high density lipoprotein; LDL: low density lipoprotein; HbA1c: hemoglobin A1c; eGFR: estimated glomerular filtration rate; UACR: urine albumin creatinine ratio; cIMT: carotid intima-media thickness.

Sex-based disparities are shown in Table 3. Multivariate logistic regression analysis of women showed that age (adjusted odds ratio [aOR] 1.076, 95% CI: 1.042–1.110) and HTN (aOR 2.089, 95% CI 1.223–3.570) were independently associated with SA but not PAU after adjusting for confounding factors (aOR 0.571, 95% CI 0.302–1.080). For men, independent factors related to SA were PAU (aOR 1.881, 95% CI 1.093–3.236), age (aOR 1.105, 95% CI 1.075–1.136), and BMI (aOR 1.149, 95% CI 1.039–1.271). After multivariate adjustment, the interaction between PAU and sex was significant (*p* = 0.005) (Figure 1D).

**Table 3:** Univariate and multivariate logistic regression analysis of factors associated with the incidence of cIMT ≥0.75 mm (75th percentile) in non-DM patients (*n* = 1228).

**Female (*n* = 655) Variable**	**Univariate analysis**			**Multivariate analysis**		
	**OR**	**95% CI**	***p* value**	**OR**	**95% CI**	***p* value**
UACR ≥30 mg/g	0.731	0.411−1.299	0.285	0.571	0.302−1.080	0.085
Age	1.096	1.071−1.123	<0.001	1.076	1.042−1.110	<0.001
BMI	1.061	1.009−1.114	0.020	1.023	0.938−1.115	0.608
Smoking	1.939	0.759−4.955	0.166			
Hypertension	2.541	1.738−3.716	<0.001	2.089	1.223−3.570	0.007
Anti-HTN medications	1.905	1.191−3.048	0.007	0.827	0.432−1.583	0.567
Dyslipidemia	1.680	0.825−3.418	0.153			
Total Cholesterol	1.001	0.996−1.006	0.695			
TG	1.003	1.000−1.007	0.059			
HDL	0.992	0.978−1.005	0.227			
LDL	1.001	0.996−1.007	0.646			
UA	1.359	1.157−1.596	<0.001	1.179	0.961−1.446	0.114
FBS	1.001	0.983−1.019	0.939	0.982	0.960−1.004	0.103
eGFR	0.953	0.938−0.968	<0.001	0.993	0.970−1.016	0.529
Metabolic syndrome	2.061	1.387−3.063	<0.001	1.486	0.824−2.678	0.188
Total body fat percentage	1.005	0.975−1.035	0.766			
**Male (*n* = 573)**						
UACR ≥30 mg/g	2.228	1.407−3.528	0.001	1.881	1.093−3.236	0.023
Age	1.087	1.065−1.109	<0.001	1.105	1.075−1.136	<0.001
BMI	1.055	1.003−1.110	0.039	1.149	1.039−1.271	0.007
Smoking	1.045	0.727−1.501	0.812			
Hypertension	2.046	1.426−2.934	<0.001	1.660	0.951−2.898	0.075
Anti-HTN medications	1.518	0.978−2.357	0.063	0.714	0.360−1.416	0.335
Dyslipidemia	1.253	0.504−3.119	0.627			
Total Cholesterol	0.998	0.993−1.004	0.521			
TG	0.998	0.996−1.000	0.080			
HDL	0.992	0.979−1.006	0.272			
LDL	1.003	0.997−1.008	0.291			
UA	1.094	0.965−1.240	0.160			
FBS	0.999	0.982−1.016	0.897			
eGFR	0.974	0.960−0.989	0.001	1.007	0.986−1.029	0.508
Metabolic syndrome	1.364	0.913−2.036	0.130			
Total body fat percentage	1.037	1.009−1.066	0.009	0.976	0.930−1.025	0.338

Abbreviations: BMI: body mass index; HTN: hypertension; TG: triglyceride; HDL: high density lipoprotein; LDL: low density lipoprotein; UA: uric acid; FBS: fasting blood sugar; eGFR: estimated glomerular filtration rate.

## DISCUSSION

The present study investigated whether an association of PAU with SA may be sex-determined in subjects without CAD, DM, or CKD. Men with PAU were older, had larger waist circumference, and exhibited a higher prevalence of HTN and metabolic syndrome than those with normal UACR. After adjusting for confounding factors, PAU was significantly associated with SA in men, but not in women.

### PAU and sex difference

We recruited healthy individuals and focused on sex-based differences in the prevalence of PAU and SA. In previous studies, age-related increases of urinary albumin excretion are more pronounced in men than in women [20, 21]. Studies of potential sex-based differences in the association of obesity and MA have reached divergent conclusions in different regions and race.

Foster et al. [22] found that visceral adipose tissue volume determined by multi-detector computed tomography was related to MA in men but not in women and the similar result was found in Japan and Singapore studies [23, 24]. In contrast, two cross-sectional studies in Chinese and Korea revealed that women with central obesity tended to develop albuminuria but insignificant relationship between albuminuria and obesity in men [25, 26]. Our data support that in men, microalbuminuria is the clusters with the components of MetS, especially central obesity more propensity than women, consistent with a finding in 408,527 UK Biobank participants that each 0.06 increase of waist-to-hip ratio was associated with 75% (71–79%) and 40% (38–43%) increases in odds of higher UACR in men and women, respectively [27]. This suggests that the long-term sequelae of obesity, fat metabolism, and fat deposition may differ between men and women.

### PAU and the risk of CVD and CIMT

The potential clinical utility of MA as a marker of endothelial dysfunction, which precedes atherosclerosis, has been suggested by studies in type I and type II DM [28, 29]. In the PREVEND study of 7579 non-diabetic, non-hypertensive individuals, MA, defined as urinary albumin excretion 20–200 mg/L, was also linked to ECG evidence of either myocardial infarction or ischemia [30].

Sex-specific associations were an important part of this study. One of the possible reasons is that numerous changes in vascular function influenced by aging and sex hormones may interact in multiple pathways to dysregulate endothelial function. During aging, lower levels of the testosterone precursor dehydroepiandrosterone (DHEA) may promote central obesity, insulin resistance, and sarcopenia, predominantly in men [31, 32]. Elevated serum PAI-1 [33], endothelin [34], von Willebrand factor [35], and oxidative stress may trigger mechanisms linking MA and SA, and the male rats with spontaneous hypertension related much of the pro-atherosclerotic markers with MA than female rats [36–39]. Another possible reason is that the impact of potential confounders on PAU and CIMT may be greater in females.

A Korean cross-sectional study of adults aged 45 to 74 years showed a significant association between high normal UACR (<30 mg/g) and CIMT >0.9 mm without sex difference [40]. Another single-center prospective 15-year follow-up study with 3128 participants reported significant sex differences in the relationship between triglycerides, smoking, physical activity, and the risk of atherosclerosis [41], UACR is not related to atherosclerosis. According to the Kidney Disease: Improving Global Outcomes working group suggests that UACR ≥30 mg/g indicates a moderate risk of CKD even in the normal eGFR, our study separated the UACR according to whether more than 30 mg/g or not. The clinical utility of MA as a standard independent risk indicator in the absence of diabetes is still debated, and the association of elevated UACR with CIMT may differ by sex; the evidence is limited. Our study demonstrated sex-based disparity in the association of PAU with SA in the relatively healthy community dwelling populations.

### Limitations

Several limitations of our study should be mentioned. First, the retrospective and observational study design could not fully assess the etiologic relationship between PAU, CVD risk factors, and SA. Second, although the measurement of albumin and creatinine from spot urine samples is straightforward, results may be less accurate than those of 24-hour urine collections. Third, confounding effects of diet, exercise, and psychological stress of 24-hour urine collections could not be excluded. Fourth, the cross-sectional cohort study lacked the long-term follow-up characteristic of longitudinal study designs; thus, we could not investigate the impact of PAU on the risk of CVD. Fifth, a potential residual confounding effect of PAU between men and women may explain the discrepant associations between UACR and SA. Sixth, the binary classification of CIMT as greater or less than the 75th percentile was an indirect assay of the severity of carotid atherosclerosis. Seventh, this study did not identify the pathogenic link between albuminuria level and the severity of atherosclerosis.

## CONCLUSION

This retrospective cohort study demonstrated a sex-based disparity in the association of PAU with SA in patients without CAD, DM, or CKD after adjusting for traditional risk factors of atherosclerosis. Our findings suggest a sex-specific risk management strategy for preventing atherosclerosis.

## METHODS

### Study population

**Figure 2:**
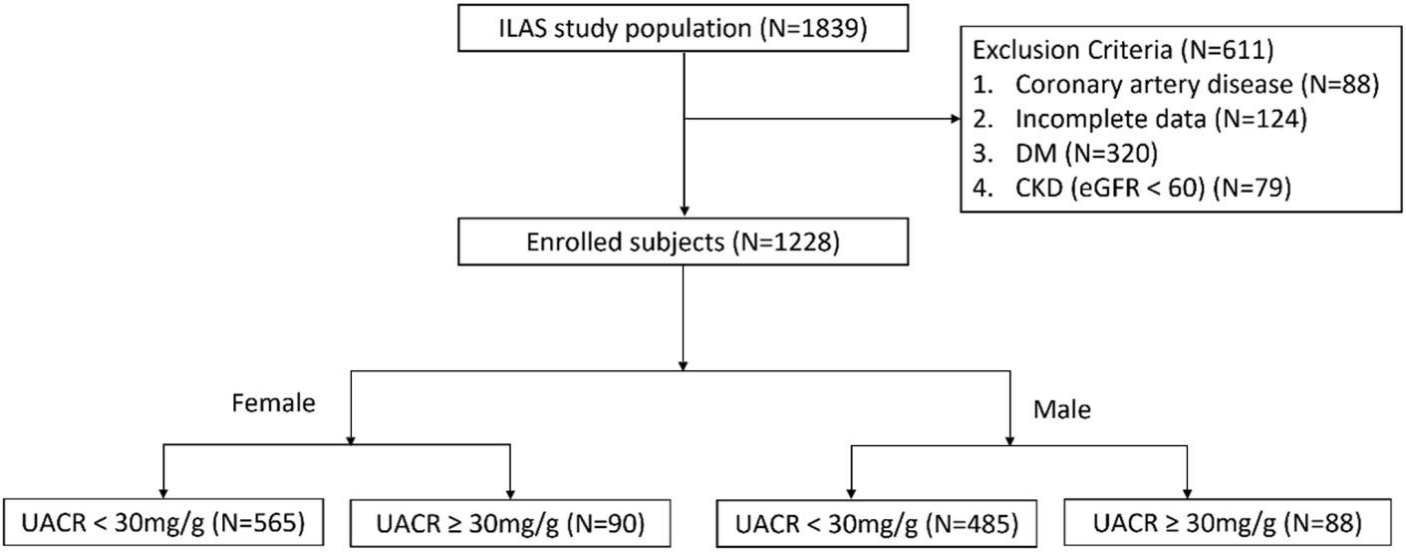
Flow chart of patient enrollment.

This retrospective cohort study was excerpted from the first wave of the I-Lan Longitudinal Aging Study (ILAS), a cohort study of community-dwelling adults aged more than 50 years who were randomly recruited through household registration records. The ILAS design, participant recruitment, and data collection have been reported previously [42]. Exclusion criteria of the present study were: (i) inability to cooperate or communicate with study investigators; (ii) declined to grant consent; (iii) currently institutionalized; comorbidities, such as active cancer, sepsis, heart failure, chronic obstructive pulmonary disease, or functional dependence; (v) life expectancy of less than six months; and (vi) planned to leave I-Lan county. A total of 1,839 community-dwelling older adults were enrolled from August 2011 to August 2013. After in-person face-to-face interviews conducted by well-trained research nurses, all participants provided written informed consent. Three hundred eighty-two subjects were excluded, including 88 with coronary artery disease, 124 with incomplete data, 320 with DM, and 79 with renal dysfunction (Figure 2). The study protocol complied with the Declaration of Helsinki and was approved by the institutional review board of the National Yang-Ming University (YM103008).

### Anthropometry, demography, and laboratory examinations

The research nurse collected the participants’ demographic and medical data, including weight, height, BMI, and waist circumference. Brachial blood pressure was measured with a mercury sphygmomanometer after the subjects rested for 15 min. Medical histories of underlying diseases, medications, and smoking were obtained from personal interviews and medical records. Peripheral blood samples were collected at 7–9 AM, after fasting for at least ten hours, to determine the concentrations of hemoglobin A1c, fasting blood glucose, total cholesterol, HDL, LDL, triglyceride, uric acid, and high-sensitivity C-reactive protein by using an automatic analyzer (ADVIA 1800; Siemens, Malvern, PA, USA).

Metabolic syndrome was defined according to the criteria proposed by Taiwan’s Ministry of Health and Welfare, with more than three of the following risk determinants: (i) waist circumference >90 cm for men or >80 cm for women; (ii) systolic blood pressure ≥130 mmHg, diastolic blood pressure ≥85 mmHg, or taking antihypertensive agents; (iii) HDL <40 mg/dL for men or <50 mg/dL for women; triglyceride ≥150 mg/dL; (iv) fasting blood glucose ≥100 mg/dL or antihyperglycemic therapy. eGFR was calculated using the Chronic Kidney Disease Epidemiology Collaboration (CKD-EPI) equation [43]. Renal dysfunction was defined as a glomerular filtration rate of <60 (mL × min^–1^ per 1.73 m^2^ body surface area) according to the 2012 KDIGO CKD classification [44].

All participants underwent whole-body dual-energy X-ray absorptiometry to measure total fat weight with a Lunar Prodigy instrument (GE Healthcare, Madison, WI, USA). Total fat percentage was defined as total fat weight divided by total body mass.

### Definition of albuminuria

A single voided morning urine sample was used to measure UACR (mg/g). UACR measured in a spot urine sample is highly correlated with 24-h urine albumin excretion [45]. UACR ≥30 mg/g was considered pathological [46]. Patients were separated into two groups whether UACR ≥30 mg/g. The flowchart of patient enrollment and classification is illustrated in Figure 2.

### Assessment of CIMT and SA

CIMT was measured using a high-resolution, broad-width, linear array transducer (LOGIQ 400 PRO; GE, Cleveland, OH, USA) at the level of the common carotid artery. All examinations were performed by the same trained technician who obtained bilateral longitudinal views of the proximal to distal sections of the common carotid arteries. The mean CIMT was defined as the average of right-and left-side CIMT values. The 75th percentile of CIMT was defined as the upper limit of normal, the threshold indicative of increased cardiovascular risk and early phase of SA [47, 48]. In the present study, the 75th percentile of CIMT was 0.75 mm.

### Statistical analysis

Data were expressed as frequencies (percentages) for categorical variables and as means ± standard deviations for continuous variables with normal distribution and median with the interquartile range due to non-normal distribution. The chi-square test was used for comparisons between two groups of categorical variables. The independent *t*-test was employed for continuous variables with normal distribution. The Kruskal-Wallis test was used for non-normally distributed continuous variables. Pearson and Spearman’s tests were used to assess the correlation between the UACR and CIMT with other variables. Associations between factors and SA were expressed as odds ratios (ORs). Multivariate logistic regression evaluated the SA by dividing both sexes after using the enter methods to explore the association between microalbuminuria and subclinical atherosclerosis and other variables. ORs with 95% confidence intervals (95% CI) for the risk of CIMT ≥0.75 mm were reported. Statistical analyses were performed using SPSS (version 22.0; IBM Corporation, Armonk, New York, NY, USA). Two-tailed *p* values <0.05 were regarded as statistically significant.

### Consent for publication

No individual participant data were reported that would require consent from the participant to publish.

### Availability of data and materials

The dataset used and analyzed during the current study is available from the corresponding author upon request.
